# Blood lead levels of Korean lead workers in 2003–2011

**DOI:** 10.1186/s40557-014-0030-3

**Published:** 2014-10-01

**Authors:** Ji-Hye Kim, Eun-A Kim, Dong-Hee Koh, Kiwhan Byun, Hyang-Woo Ryu, Sang-Gil Lee

**Affiliations:** 1 Occupational Safety and Health Research Institute, Korea Occupational Safety and Health Agency, Jongga-ro 400, Jung-gu, Ulsan 681-230, Republic of Korea; 2 National Cancer Center, Ilsan-ro 323, Ilsandong-gu, Goyang-city 410-769, Republic of Korea

**Keywords:** Blood lead level, Lead worker, Special health examination, Korean

## Abstract

**Objectives:**

This study aimed to document the trend in blood lead levels in Korean lead workers from 2003 until 2011 and blood lead levels within each of the main industries.

**Methods:**

Nine years (2003–2011) of blood lead level data measured during a special health examination of Korean lead workers and collected by the Korea Occupational Safety and Health Agency were analyzed. Blood lead levels were determined by year, and a geometric mean (GM) was calculated for each industry division.

**Results:**

The overall GM blood lead level for all years combined (n = 365,331) was 4.35 μg/dL. The GM blood lead level decreased from 5.89 μg/dL in 2003 to 3.53 μg/dL in 2011. The proportion of the results ≥30 μg/dL decreased from 4.3% in 2003 to 0.8% in 2011. In the “Manufacture of Electrical Equipment” division, the GM blood lead level was 7.80 μg/dL, which was the highest among the industry divisions. The GM blood lead levels were 7.35 μg/dL and 6.77 μg/dL in the “Manufacturers of Rubber and Plastic Products” and the “Manufacture of Basic Metal Products” division, respectively.

**Conclusions:**

The blood lead levels in Korean lead workers decreased from 2003 to 2011 and were similar to those in the US and UK. Moreover, workers in industries conventionally considered to have a high risk of lead exposure also tended to have relatively high blood lead levels compared to those in other industries.

## 1 Introduction

Lead is a soft, malleable, blue-gray heavy metal characterized as having a high density and being resistant to corrosion [1]. Because of lead’s chemical and physical properties including a high density, softness, a low melting point, resistance to corrosion, and opacity to gamma and x-rays, lead and its compounds are used in a wide variety of industrial applications [2]. For example, lead is used to manufacture storage batteries, alloys, cables, polyvinyl chloride (PVC) stabilizers, paints, crystal, ceramics, and bullets, among other things [3]. Lead is also used in the construction for attenuation of sound and vibration and for radioactive shielding [1].

However, lead serves no useful biologic function in humans, and lead exposure can result in acute or chronic adverse effects in multiple organ systems [1]. Recent research has raised concerns regarding the toxicity of lead at the blood lead levels as low as 5 μg/dL [4],[5]. Since occupational lead exposure first came to the public’s attention in 1967 in Korea, subsequent progress has been made in lowering workplace lead exposure and implementing large-scale health screenings [6]. Since 1972, all workers with lead exposure are placed under medical surveillance according to the Industrial Safety and Health Act, which requires the blood lead concentration of these workers to be measured at least once every year.

A new biological monitoring program that used blood lead and zinc protoporphyrin levels and a respiratory protection program that were introduced in the 1990s are considered to have contributed to the considerable decrease in lead absorption in lead workers [6]. However, to the best of our knowledge, no longitudinal analysis of blood lead levels in Korean lead workers has occurred on a national scale since the 2000s. Thus, the aims of this investigation were to document the trend in blood lead levels in Korean lead workers from 2003 until 2011 and blood lead levels within each of the main industries.

## 2 Materials and methods

The Korea Occupational Safety and Health Agency have collected data of the special health examination from all occupational health institutes in Korea under the Industrial Safety and Health Act. We acquired data over nine years (2003–2011) on Korean workers who were exposed to the following hazardous substances: lead (inorganic dust and fumes), tetraalkyl lead, lead wire, and paint containing white lead. We finally selected data for 365,331 blood lead measurements after excluding missing data and outliers (blood lead level >400 μg/dL) or values that were strongly considered to have been reported incorrectly. The number of measurements does not indicate the total number of workers because each worker may have measured their blood lead concentration more than once per year.

The limit of detection was set at 0.85 μg/dL [7], and all results <0.85 μg/dL or reported as “not detected” were substituted using the equation $\sqrt{1/3\times 0.85}\text{,}$ since special occupational health institutes have various capacities for the detection of lead. Then, the geometric mean (GM) blood lead levels in each year were determined.

The blood lead levels were divided into seven categories based on their health effects and regulatory criteria as follows: <3 μg/dL, 3 μg/dL to <5 μg/dL, 5 μg/dL to <10 μg/dL, 10 μg/dL to <30 μg/dL, 30 μg/dL to <40 μg/dL, 40 μg/dL to <60 μg/dL, and ≥60 μg/dL. The US Centers for Disease Control and Prevention designated 10 μg/dL as the reference blood lead level for adults [8]. In Korea, 30 μg/dL and 40 μg/dL are the criteria for the primary observation and diagnosis of lead poisoning, respectively. Under the US Occupational Safety and Health Administration lead standard for General Industry, workers with a blood lead level ≥60 μg/dL are required to be removed immediately from lead exposure [9].

We also analyzed the GM blood lead levels of workers according to their type of industry. Errors in the industrial classifications made by several companies were detected; therefore, not all industry divisions were included. Industry divisions with a high risk for lead exposure [10] or a total number of measurements >1,000 were selected for analysis. In addition, three industry sub-classes (“Manufacture of Accumulators”, “Manufacture of Smelting, Refining and Alloys of Lead, Zinc and Tin”, and “Manufacture of Inorganic Pigments and Other Metal Oxides”) which cover conventional high risk industries for lead exposure, were included. The ninth edition of Korean Standard Industrial Classification (KSIC-9) [11], which classifies all industries into 21 sections, 76 divisions, 228 groups, 487 classes, and 1,145 sub-classes was followed. The KSIC codes from the eighth edition were converted into the codes for the ninth edition using their conversion table.

All analyses were performed using SAS (version 9.2; SAS Institute Inc., Cary, NC, USA). Two-sample t-test was performed to evaluate difference in blood lead levels between genders.

This study was reviewed for private information protection and approved by the Institutional Review Board of the Occupational Safety and Health Research Institute.

## 3 Results

The GM blood lead level for the entire sample of measurements (n = 365,331) was 4.35 μg/dL with 310,724 measurements from male workers (GM = 4.62 μg/dL) and 54,607 measurements from female workers (GM = 3.09 μg/dL). The difference in GM between genders was significant (p<0.001) (Table 1).

**Table 1:** Blood lead levels in Korean lead workers measured during a special health examination (2003–2011)

	**n (%)**	**Age (years; mean ± SD)**	**BLL (μg/dL; GM (95% CI))**	p-value*
**Total**	365331 (100)	37.8 ± 9.9	4.35 (4.34-4.36)	
**Men**	310724 (85.0)	38.2 ± 9.7	4.62 (4.61-4.63)	< 0.001
**Women**	54607 (14.9)	35.4 ± 10.6	3.09 (3.07-3.11)	

*determined using a *t*-test.

SD, standard deviation; BLL, blood lead level; GM, geometric mean; CI, confidence interval.

**Figure 1:**
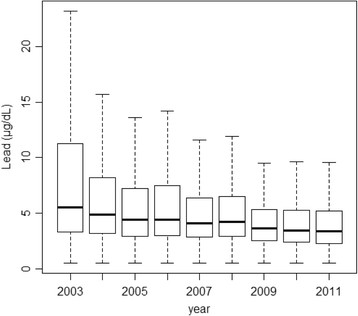
**Median blood lead levels of Korean lead workers from 2003 to 2011.** The values are reported as the 25th, 50th, and 75th percentiles for each year.

There was a decreasing trend in the GM (2003, 5.89 μg/dL; 2011, 3.53 μg/dL; Table 2) and median (2003, 5.50 μg/dL; 2011, 3.38 μg/dL; Figure 1) blood lead levels over time. The total number of measurements (2003, 31,137; 2011, 49,772) and the mean age increased over time. The proportion of male workers was considerably higher than that of female workers, and the proportion of male workers increased over time (2003, 75.2%; 2011, 88.6%).

**Table 2:** Blood lead levels in Korean lead workers by year

**Year**	**n**	**Men (%)**	**Women (%)**	**Age (years; mean ± SD)**	**BLL (μg/dL; GM (95% CI))**
**2003**	31137	23402 (75.2)	7735 (24.8)	35.4 ± 9.7	5.89 (5.83-5.95)
**2004**	34701	27317 (78.7)	7384 (21.3)	36.7 ± 10.1	5.22 (5.17-5.27)
**2005**	33312	26832 (80.5)	6480 (19.5)	37.2 ± 10.1	4.62 (4.58-4.66)
**2006**	43899	37289 (84.9)	6610 (15.1)	37.1 ± 9.7	4.98 (4.94-5.01)
**2007**	43517	37650 (86.5)	5867 (13.5)	37.6 ± 9.8	4.42 (4.39-4.45)
**2008**	42866	37470 (87.4)	5396 (12.6)	38.3 ± 9.8	4.39 (4.35-4.42)
**2009**	41745	37285 (89.3)	4460 (10.7)	39.1 ± 9.9	3.77 (3.74-3.79)
**2010**	44382	39361 (88.7)	5021 (11.3)	38.9 ± 9.7	3.63 (3.60-3.65)
**2011**	49772	44118 (88.6)	5654 (11.4)	38.5 ± 9.7	3.53 (3.50-3.55)

SD, standard deviation; BLL, blood lead level; GM, geometric mean; CI, confidence interval.

**Figure 2:**
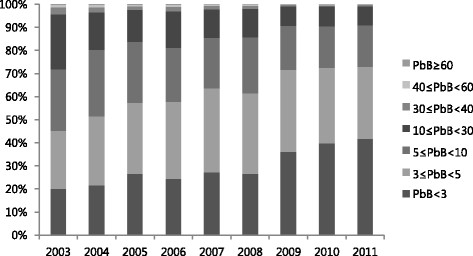
Distribution of blood lead level (μg/dL) measurements by category and year.

The distribution by blood lead level by category and year is shown in Table 3 and Figure 2. A substantial proportion of blood lead level measurements was <5 μg/dL in 2003 (45.34%), and the proportion of measurements <5 μg/dL increased over time (2011, 72.94%). In addition, <5% of all annual measurements were ≥30 μg/dL, and <1% measurements were ≥60 μg/dL.

**Table 3:** Distribution of blood lead level (μg/dL) measurements by each exposure category and year

**Year**	**PbB <3 μg/dL**	**3**≤ **PbB <5**	**5**≤ **PbB <10**	**10**≤ **PbB <30**	**30**≤ **PbB <40**	**40**≤ **PbB <60**	**PbB** ≥**60**	**Total**
**2003**	6269 (20.13)	7849 (25.21)	8223 (26.41)	7467 (23.98)	918 (2.95)	368 (1.18)	43 (0.14)	31137
**2004**	7471 (21.53)	10440 (30.09)	9971 (28.73)	5618 (16.19)	749 (2.16)	410 (1.18)	42 (0.12)	34701
**2005**	8826 (26.49)	10319 (30.98)	8757 (26.29)	4672 (14.02)	446 (1.34)	245 (0.74)	47 (0.14)	33312
**2006**	10666 (24.30)	14696 (33.48)	10266 (23.39)	6924 (15.77)	877 (2.00)	426 (0.97)	44 (0.10)	43899
**2007**	11897 (27.34)	15819 (36.35)	9519 (21.87)	5374 (12.35)	632 (1.45)	259 (0.60)	17 (0.04)	43517
**2008**	11448 (26.71)	14931 (34.83)	10348 (24.14)	5352 (12.49)	537 (1.25)	224 (0.52)	26 (0.06)	42866
**2009**	15070 (36.10)	14815 (35.49)	8015 (19.20)	3513 (8.42)	234 (0.56)	82 (0.20)	16 (0.04)	41745
**2010**	17699 (39.88)	14502 (32.68)	7959 (17.93)	3820 (8.61)	252 (0.57)	130 (0.29)	20 (0.05)	44382
**2011**	20807 (41.80)	15498 (31.14)	8953 (17.99)	4095 (8.23)	340 (0.68)	76 (0.15)	3 (0.01)	49772
**Total**	110153 (30.15)	118869 (32.54)	82011 (22.45)	46835 (12.82)	4985 (1.36)	2220 (0.61)	258 (0.07)	365331

Values reported as n (%). PbB, blood lead concentration (μg/dL).

The number of measurements that fell within the criterion for primary observation in Korea (≥30 μg/dL) decreased from 2003 (n = 1,329, 4.27%) to 2011 (n = 419, 0.84%) as did the number of measurements that indicated a diagnosis of lead poisoning (≥40 μg/dL) (2003, n = 411, 1.32%; 2011, n = 79, 0.16%).

The GM blood lead levels were 7.80 μg/dL in the “Manufacture of Electrical Equipment” division, which includes the manufacture of storage batteries (accumulators), 7.35 μg/dL in the “Manufacture of Rubber and Plastic Products” division and 6.77 μg/dL in the “Manufacture of Basic Metal Products” division, which is considered to have a high risk for lead exposure. The GM blood lead levels were 6.27 μg/dL in the “Manufacture of Other Non-metallic Mineral Products” division, 5.93 μg/dL in the “Manufacture of Chemical and Chemical Products Except Pharmaceuticals and Medicinal Chemicals” division, and 5.03 μg/dL in the “Manufacture of Fabricated Metal Products, Except Machinery and Furniture” division. The largest proportion of measurements was documented in the “Manufacture of Electronic Components, Computer, Radio, Television and Communication Equipment and Apparatuses” division (n = 69,587); however, the GM blood lead level (3.24 μg/dL) was the lowest of the manufacturing divisions (Table 4).

**Table 4:** Geometric means of blood lead levels by Korean industry division (Manufacturing; 2003–2011)

**KSIC-9 code**	**Industry division**	**n**	**GM (μg/dL; 95% CI)**
28	Manufacture of Electrical Equipment	35389	7.80 (7.71-7.89)
22	Manufacture of Rubber and Plastic Products	7149	7.35 (7.18-7.52)
24	Manufacture of Basic Metal Products	32279	6.77 (6.70-6.84)
23	Manufacture of Other Non-metallic Mineral Products	5639	6.27 (6.14-6.40)
20	Manufacture of Chemicals and Chemical Products, Except Pharmaceuticals and Medicinal Chemicals	16712	5.93 (5.84-6.02)
25	Manufacture of Fabricated Metal Products, Except Machinery and Furniture	9904	5.03 (4.95-5.11)
29	Manufacture of Other Machinery and Equipment	16217	3.79 (3.75-3.83)
27	Manufacture of Medical, Precision and Optical Instruments, Watches and Clocks	9346	3.63 (3.58-3.69)
30	Manufacture of Motor Vehicles, Trailers, and Semitrailers	17334	3.58 (3.53-3.62)
31	Manufacture of Other Transport Equipment	53463	3.57 (3.55-3.58)
26	Manufacture of Electronic Components, Computer, Radio, Television and Communication Equipment and Apparatuses	69587	3.24 (3.22-3.26)
Total		273019	4.50 (4.48-4.51)

KSIC-9, Korean Standard Industrial Classification; GM, geometric mean; CI, confidence interval.

The GM blood lead levels in workers in the three industry sub-classes, “Manufacture of Smelting, Refining and Alloys of Lead, Zinc and Tin” (KSIC-9 code 24213, n = 4,551), “Manufacture of Accumulators” (KSIC-9 code 28202, n = 18,930), and “Manufacture of Inorganic Pigments and Other Metal Oxides” (KSIC-9 code 20131, n = 2,509), were 18.81 μg/dL (95% CI = 18.53–19.10), 16.28 μg/dL (95% CI = 16.15–16.41), and 9.26 μg/dL (95% CI = 8.86–9.67), respectively.

In the non-manufacturing sector, the “Special Trade Construction”, “Waste Collection, Disposal, and Materials Recovery”, and “Wholesale Trade and Commission Trade, Except Motor Vehicles and Motorcycles” divisions had relatively high GM blood lead levels (6.07 μg/dL, 5.58 μg/dL, and 4.98 μg/dL, respectively; Table 5).

**Table 5:** Geometric means of blood lead levels by Korean industry division (Non-manufacturing; 2003–2011)

**KSIC-9 code**	**Industry division**	**n**	**GM (μg/dL; 95% CI)**
42	Special Trade Construction	2201	6.07 (5.88-6.27)
38	Waste Collection, Disposal and Materials Recovery	1093	5.58 (5.27-5.92)
46	Wholesale Trade and Commission Trade, Except Motor Vehicles and Motorcycles	1321	4.98 (4.76-5.22)
96	Other Personal Services Activities	3168	4.71 (4.58-4.84)
84	Public Administration and Defense; Compulsory Social Security	2211	4.32 (4.16-4.48)
75	Business Support Services	3502	4.29 (4.17-4.42)
61	Telecommunications	9267	4.19 (4.13-4.25)
41	General Construction	1615	4.08 (3.94-4.23)
95	Maintenance and Repair Services	38864	4.04 (4.02-4.06)
63	Information Service Activities	1686	3.93 (3.84-4.01)
52	Storage and Support Activities for Transportation	1542	3.61 (3.48-3.74)
86	Human Health	3106	3.02 (2.95-3.10)
49	Land Transport; Transport Via Pipelines	10213	2.74 (2.71-2.77)
Total		79789	3.92 (3.90-3.94)

KSIC-9, Korean Standard Industrial Classification; GM, geometric mean; CI, confidence interval.

## 4 Discussion

The findings of this study demonstrated that the blood lead levels in Korean lead workers decreased from 2003 to 2011 (Table 2). A similar trend was found in the general Korean population. The Korean National Health and Nutrition Examination Survey (KNHANES) measured the concentrations of heavy metals in the blood since 2005. According to the KNHANES, the GM blood lead levels in adults (>19 years old) in 2005 and 2008 were 2.66 μg/dL [12] and 2.32 μg/dL (men, 2.76 μg/dL; women, 1.96 μg/dL) [13], respectively. Moreover, this trend continued until 2011 (2009, 2.29 μg/dL [14]; 2010, 2.24 μg/dL [15]; and 2011, 2.15 μg/dL [16]). The GM blood lead levels in our analysis also tended to decrease from 2005 (4.62 μg/dL) to 2011 (3.53 μg/dL); however, these values are still higher than those in general population are.

Similar trends have been observed in lead surveillances in other countries. In the US, the National Institute for Occupational Safety and Health has conducted the Adult Blood Lead Epidemiology and Surveillance program since 1987. This program tracks laboratory reported elevated blood lead levels, which were originally defined as blood lead levels ≥25 μg/dL and later changed to blood lead levels ≥10 μg/dL in 2009 [8]. The results of the 2008–2009 analysis indicated that the prevalence of elevated blood lead levels (≥25 μg/dL) decreased from 14.0 per 100,000 employed adults in 1994 to 6.3 per 100,000 employed adults in 2009. Further, the prevalence of adults with blood lead levels ≥40 μg/dL decreased from 3.5 per 100,000 employed adults in 1994 to 0.9 per 100,000 employed adults in 2009 [17].

In the UK, the Health and Safety Executive (HSE) is responsible for collecting and analyzing UK lead surveillance data. The number of men with blood lead levels ≥40 μg/dL decreased from 2,367 (15.4% of male workers) in 2000–2001 to 524 (7.6% of male workers) in 2009–2010; this change was also reflected in a decrease in the median blood lead level range from 20–24 μg/dL to 10–19 μg/dL, respectively. Similarly, the number of women with blood lead levels ≥40 μg/dL decreased from 7 (1% of female workers) in 2000–2001 to 0 in 2009–2010, and the median blood lead level was <10 μg/dL at both time intervals [18]. Comparatively, the blood lead levels of lead workers in the present study were lower (2003, GM = 5.89 μg/dL; 2011, GM = 3.53 μg/dL) than those reported by the HSE. Furthermore, the number of measurements ≥40 μg/dL was 411 (1.32% of the total sample) in 2003 and 79 (0.16% of the total sample) in 2011 in our analysis.

Differences in the industrial structure, workplace lead exposure, and exposure control may explain these differences between the countries. However, it might also be possible that Korean workers are monitored more extensively than they are in other countries since we do not require a specific lead exposure level to initiate biological monitoring. In the US, Occupational Safety and Health Standards established an action level of 30 μg/m^3^, a time-weighted average, based on an 8-hour work-day, to initiate medical surveillance [9]. Nevertheless, employers might not provide blood lead level testing to all lead-exposed workers as required by Occupational Safety and Health Administration regulations [19],[20].

Under the Control of Lead at Work Regulations (CLAW) in the UK, all workers with significant exposure to lead are required to undergo medical surveillance. However, the decision to place workers under surveillance rests with the employer [21]. There has been a recent reduction in the overall number of British workers under medical surveillance for work-related lead exposure, a 56% decrease from 16,127 in 2000–2001 to 7,162 in 2009–2010 [18]. In the present study, blood lead levels <5 μg/dL comprised >50% of the total sample; however, in the HSE report, only 20%–40% of the results were <10 μg/dL [18]. Therefore, we suggest that the UK lead surveillance primarily involves workers at a high risk for lead exposure. However, in Korean, lead surveillance tends to involve workers with very low blood lead concentrations as well as high risk workers because no clear inclusion criteria for special health examinations exist, and many low risk workers are assumed included.

In the comparison analysis by industry, we found “Manufacture of Electrical Equipment” division to have the highest GM blood lead level (7.80 μg/dL). This could be attributed to lead exposure in the manufacture of lead storage batteries. The GM blood lead level in the “Manufacture of Accumulators” sub-class was 16.28 μg/dL. In a 1998 study of 1,782 Korean storage battery workers from eight facilities, the arithmetic mean blood lead level was 31.0 μg/dL, and 21.89% of the workers had a blood lead level ≥40 μg/dL [22]. High dose exposure is more likely to occur in small plants where workplace exposures tend to be poorly controlled compared to larger plants [23].

The GM blood lead level in the “Manufacture of Rubber and Plastic Products” division was 7.35 μg/dL. A cross-sectional analysis of blood lead levels in Korean lead workers in 2003 also demonstrated that lead workers in plastic industries are at a very high risk of severe lead poisoning [24]. In particular, the process of manually mixing stabilizers while manufacturing PVC products may lead to high doses of lead exposure.

Primary and secondary smelting is another process in which the risk of poisoning is high, partly from the lead fumes generated at the necessary high temperatures and partly from the lead oxide dust spread around the smelter, and control of this risk has proven to be difficult [25]. In 1999, the arithmetic mean blood lead level of 233 primary smelting workers was 26.7 μg/dL and that of 32 secondary smelting workers was 50.3 μg/dL [26]. The GM blood lead level in the “Manufacture of Smelting, Refining and Alloys of Lead, Zinc and Tin” sub-class was 18.81 μg/dL in our study.

In the “Manufacture of Chemicals and Chemical Products Except Pharmaceuticals and Medical Chemicals” division, which includes the manufacture of lead compounds (e.g. litharge (PbO), minium (Pb_3_O_4_), and PVC stabilizer), the GM blood lead level was 5.93 μg/dL. In addition, the GM blood lead level was 9.26 μg/dL in the “Manufacture of Inorganic Pigments and Other Metal Oxides” sub-class. In a study conducted in 1997, the arithmetic mean blood lead level of 120 litharge manufacturing workers was 58.8 μg/dL, and, in a study conducted in 1999, the arithmetic mean blood lead level of 51 litharge manufacturing workers was 36.4 μg/dL [26],[27].

Large amounts of lead are used to manufacture electronic apparatuses, and many workers are involved in that industry. In our study, “Manufacture of Electronic Components, Computer, Radio, Television and Communication Equipment and Apparatuses” division represented the largest percentage of measurements, but it had the lowest blood lead levels (3.24 μg/dL) of all of the manufacturing divisions. In this division, many workers work as solderers, and this job is considered to have a low risk of lead exposure [23].

The “Special Trade Construction” division had a relatively high GM blood lead level in our study (6.07 μg/dL). In the US, lead standards are applied more strictly to construction workers (workers should be removed from lead exposure when blood lead levels are ≥50 μg/dL), and this reflects the hazardous workplace in the construction industry.

The “Waste Collection, Disposal, and Materials Recovery” division had a GM blood lead level of 5.58 μg/dL and includes the “Recovery of Metal and Non-Metal Waste and Scrap” group as an industrial group, which is conventionally regarded as an industry with high lead exposure. A previous study reported that the industry classes of “Metal Waste and Scrap” and “Non-Metal Waste and Scrap” had GM blood lead levels of 17.4 μg/dL and 24.2 μg/dL, respectively [24]. Welding or cutting structures composed of lead or covered with lead paint, such as during the demolition of old ships, requires high temperatures and would generate high concentrations of lead fumes [23].

The “Wholesale Trade and Commission Trade, Except Motor Vehicles and Motorcycles” division includes wholesale of materials for recycling and retail businesses combined with repair services. The risk of lead exposure would appear to be low in this industry; however, the GM blood lead level was 4.98 μg/dL, which is relatively high compared to that in the manufacturing divisions.

To the best of our knowledge, this is the first study to document the temporal trend of blood lead levels in Korean lead workers on a national basis using data from 2000s. Our analysis comes after the improvements in the control of lead exposure and surveillance of blood lead concentrations that occurred in the 1990s and contributed to a decrease in the blood lead levels in Korean workers at that time. In addition, we included the entire population of Korean lead workers in our analysis. Previous studies have primarily focused on lead exposure within specific workplaces. In our analysis, we found that blood lead levels tended to decrease in Korean lead workers between 2003 and 2011, and workers in industries that are conventionally considered at high risk for lead exposure had relatively higher blood lead levels than others did. A temporal decrease in blood lead levels has also been reported in the general Korean population and lead workers in other countries. This recent reduction in blood lead levels in Korean lead workers, in part, might be attributable to a decrease in lead in general population. This finding suggests the usefulness of public health measures as well as occupational health measures in reducing lead exposure. From an occupational health perspective, these improvements seem to be related to improved protection from occupational lead exposure and strengthened regulations and standards. The number of measurements that were ≥ 10 μg/dL decreased from 2003 (n=8,796) to 2011 (n=4,514) as did the number of measurements that were ≥ 30 μg/dL (2003, n=1,329; 2011, n=419). This reduction might have resulted from improvements made in workplace environments. The criteria for primary observation and the diagnosis of lead poisoning were made more conservative on January 1, 2009 (from 40 and 60 μg/dL to 30 and 40 μg/dL) in Korea.

However, our study has certain limitations. The blood lead concentrations were collected from many different special occupational health institutes, and their capacities for the measurement and detection of lead varied. The limits of detection of each institute should be considered, but only one value was used for practical reason. In addition, workers may have had their blood lead concentrations measured more than once per year and which would have affected our results. In addition, not all industry sub-classes were included because of misclassifications and low reliability intensifying with increase of classification stage. Industrial misclassifications by several companies were detected; however, we were not able to check all of the classifications in this large data set. Despite these limitations, the present study is meaningful because it provides evidence of a decreasing trend of blood lead levels in Korean lead workers during the 2000s. Further studies of lead exposure in Korean lead workers and medical lead surveillance are warranted.

## Competing interests

The authors declare that they have no competing interests.

## Authors’ contributions

JHK, EAK, and SGL designed the study. DHK, KB, and HWR participated in the acquisition and analysis of data. JHK analyzed the data and drafted the manuscript. JHK, EAK, and SGL discussed the results and revised the manuscript. All authors read and approved the final manuscript.
